# The Nursing at Woodilee Asylum

**Published:** 1904-10-15

**Authors:** 


### Editor's Letter-Box.

[Our Correspondents are reminded that prolixity is a great bar to publication, and that brevity of style and conciseness of statement greatly facilitate early insertion.]

### THE NURSING AT WOODILEE ASYLUM.

Dr. Hamilton C. Marr, Medical Superintendent of the Woodilee Asylum, writes: With respect to your remarks on the 46th annual report of the Commissioners of Lunacy for Scotland in the current number of The Hospital it may be pointed out that a very erroneous conclusion can be drawn from your statement with reference to Woodilee Asylum. In commenting on the proportion of day attendants and night attendants you say that you would like to ask the Commissioners whether they consider such a proportion necessary, and, if not, whether they consider it justifiable in a rate-supported asylum. Without entering fully into the general question of the requisite proportion of attendants to patients I may point out that our Glasgow asylums are essentially different from other asylums in the country in that the hours of duty of the attendants and nurses have been markedly reduced within the last year or two. Day nurses and attendants were formerly on duty from six o'clock in the morning till eight o'clock in the evening. Instead of the 14 hours on duty thus required by the decree of the Local District Lunacy Board, which is elected by the ratepayers, the hours of duty of nurses and attendants have been reduced to an average of nine and a half hours per day; it is obvious that a larger staff of nurses and attendants is required in these circumstances. This is the whole explanation of the apparently large proportion of attendants and nurses to patients.

